# Colistin Combined With Tigecycline: A Promising Alternative Strategy to Combat *Escherichia coli* Harboring *bla*_NDM–__5_ and *mcr-1*

**DOI:** 10.3389/fmicb.2019.02957

**Published:** 2020-01-08

**Authors:** Yu-Feng Zhou, Ping Liu, Chuan-Jian Zhang, Xiao-Ping Liao, Jian Sun, Ya-Hong Liu

**Affiliations:** ^1^National Risk Assessment Laboratory for Antimicrobial Resistance of Animal Original Bacteria, College of Veterinary Medicine, South China Agricultural University, Guangzhou, China; ^2^Guangdong Provincial Key Laboratory of Veterinary Pharmaceutics Development and Safety Evaluation, South China Agricultural University, Guangzhou, China; ^3^Jiangsu Co-innovation Center for the Prevention and Control of Important Animal Infectious Disease and Zoonosis, Yangzhou University, Yangzhou, China

**Keywords:** carbapenem-resistant Enterobacteriaceae, carbapenem-resistance, colistin-resistance, combination therapy, MCR-1, New Delhi metallo-β-lactamases-5

## Abstract

Infections due to carbapenem-resistant NDM-producing *Escherichia coli* represent a major therapeutic challenge, especially in situations of pre-existing colistin resistance. The aim of this study was to investigate combinatorial pharmacodynamics of colistin and tigecycline against *E. coli* harboring *bla*_NDM–__5_ and *mcr-1*, with possible mechanisms explored as well. Colistin disrupted the bacterial outer-membrane and facilitated tigecycline uptake largely independent of *mcr-1* expression, which allowed a potentiation of the tigecycline-colistin combination. A concentration-dependent decrease in colistin MIC and EC_50_ was observed with increasing tigecycline levels. Clinically relevant concentrations of colistin and tigecycline combination significantly decreased bacterial density of colistin-resistant *E. coli* by 3.9 to 6.1-log_10_ cfu/mL over 48 h at both inoculums of 10^6^ and 10^8^ cfu/mL, and were more active than each drug alone (*P* < 0.01). Importantly, colistin and tigecycline combination therapy was efficacious in the murine thigh infection model at clinically relevant doses, resulting in >2.0-log_10_cfu/thigh reduction in bacterial density compared to each monotherapy. These data suggest that the use of colistin and tigecycline combination can provide a therapeutic alternative for infection caused by multidrug-resistant *E. coli* that harbored both *bla*_NDM–__5_ and *mcr-1*.

## Introduction

Infections caused by carbapenem-resistant Enterobacteriaceae (CRE), especially the New Delhi metallo-β-lactamases (NDM)-producing *Escherichia coli*, have become a global therapeutic challenge in clinical and public health settings (Perez and Bonomo, 2018). In general, isolates carrying *bla*_NDM_ tend to carry other resistance genes thus limiting treatment options (Falagas et al., 2014; Liu et al., 2019). Currently, the polymyxin antibiotics (polymyxin B and colistin) have reemerged as the last-line therapy against CRE. However, the clinical efficacy of polymyxin antibiotics has been significantly compromised by the widespread emergence of mobile colistin resistance gene *mcr-1* (Liu et al., 2016). Worryingly, the MCR-1-producing *E. coli* that coexist with NDM-1, NDM-5, and NDM-9 have been recently reported worldwide, and these isolates possess resistance to fluoroquinolones, sulfonamides, β-lactams, tetracycline, and aminoglycosides (Du et al., 2016; Yao et al., 2016). Fortunately, the level of *mcr-1*-mediated colistin resistance is moderate (Sun et al., 2018), thus the use of colistin-based combinations would be of considerable clinical significance.

Tigecycline is the first of glycylcycline class that exhibited mainly bacteriostatic activity (Meagher et al., 2005). Of note, the decreased clinical efficacy and increased mortality have been previously reported regarding tigecycline monotherapy in the treatment of severe infections (Yahav et al., 2011). Therefore, clinicians should avoid tigecycline monotherapy to reserve it as another last-resort drug.

**FIGURE 1:**
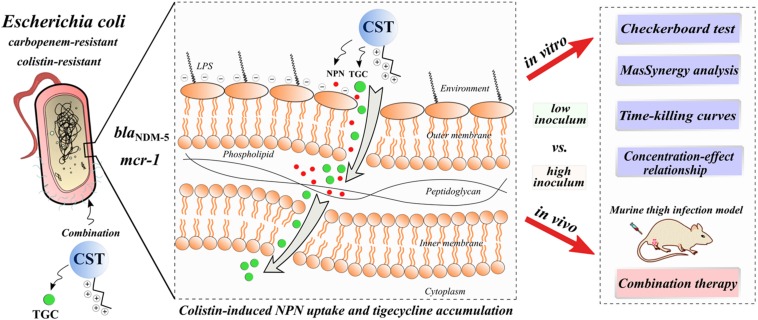
Graphic potential mechanisms for increased activity of colistin in combination with tigecycline against *E. coli* harboring *bla*_NDM–5_ and *mcr-1*: colistin-induced bacterial outer-membrane perturbation and tigecycline accumulation. CST, colistin; TGC, tigecycline.

In this study, we systemically investigated the activity of colistin and tigecycline combination at the clinically achievable concentrations *in vitro* and in a murine thigh infection model against carbapenem-resistant *E. coli* harboring *bla*_NDM–__5_, especially in situations of pre-existing the *mcr-1* gene and high bacterial burdens. Additionally, we explored the underlying mechanisms of this combination (Figure 1) by determination of bacterial out-membrane integrity and tigecycline accumulation.

## Materials and Methods

### Organisms, Media, and Antibiotics

Five well-described *E. coli* strains used in this study were 2630 (ST3902, *bla*_NDM–__5_), 3112 (ST1011, *mcr-1*), 1320 (ST648; *bla*_NDM–__5_, *mcr-1*), 2610 (ST101; *bla*_NDM–__5_, *mcr-1*), and 2121 (ST156; *bla*_NDM–__5_, *mcr-1*) (Sun et al., 2016a, b; Zhou et al., 2017). The *E. coli* strain ATCC 25922 (ST73) served as the negative control. The organisms were grown, subcultured, and quantified in cation-adjusted Mueller-Hinton broth (CAMHB) and agar (MHA; Difco Laboratories, Detroit, MI, United States). Colistin (CST), tigecycline (TGC), and other used antibiotics were purchased from Sigma-Aldrich (Shanghai, China) and prepared as fresh stock solutions in sterile water or medium prior to experiments.

### Combinatorial Susceptibility Testing

The MICs of colistin for each *E. coli* strain were determined in the absence and presence of twofold increasing tigecycline concentrations (0.13–0.5 mg/L) using a modified broth microdilution method (Wiegand et al., 2008). The interaction of this combination was evaluated in duplicate for each isolate with a checkerboard assay (CST range 0.25–32 mg/L; TGC range 0.015–32 mg/L). Inhibition was read visually to calculate the fractional inhibitory concentration index (FICI), with an FICI ≤ 0.5 deemed synergistic. In addition, cell density was assessed using a spectrometer to estimate cell densities for MacSynergy II analysis (Prichard and Shipman, 1990). The MacSynergy II program uses the Bliss independence algorithm to generate a 3-dimensional response profile of the synergy-antagonism landscape by representing the theoretical indifferent surface. Peaks and troughs represent synergy and antagonism, respectively, and the extents of these were defined using interaction volumes (μM^2^): <25, additive; 25 to 50, minor but significant; 50 to 100, moderate; and >100, strong synergy or antagonism (Deshpande et al., 2016; Lai et al., 2016). The results were expressed as the mean interaction volumes calculated at the 95% confidence level from three independent experiments.

### Assessment of Colistin-Induced Outer-Membrane Disruption

The 1-*N*-phenylnaphthylamine (NPN) assay was performed to assess bacterial outer-membrane permeability to colistin as previously described (Buyck et al., 2012). Uptake of NPN by *E. coli* cells was a measure of the degree of permeability, and the subsequent fluorescence indicated a permeability breakdown (Macnair et al., 2018). Thus, NPN uptake was used to quantitatively indicate the colistin-induced outer membrane disruption. Mid-logarithmic cultures of *E. coli* strains were washed and suspended in PBS to a density of 10^9^ cfu/mL (i.e., OD_600__nm_ = 1.0). Bacterial cells were added to PBS containing NPN (10 μM) and varying concentrations of colistin in black 96-well microplates. After 1 h of incubation at 37°C, fluorescence was read using an EnSight multimode plate reader (PerkinElmer, Waltham, MA, United States) at 355 nm excitation and 405 nm emission wavelengths. NPN uptake (%) was calculated for each *E. coli* strain as described elsewhere (Macnair et al., 2018). Full NPN uptake (100%) was achieved by adding 100 mg/L of colistin.

### Intracellular Accumulation of Tigecycline

The levels of tigecycline accumulation by *mcr-1*-positive and -negative *E. coli* strains in the absence and presence of colistin were determined as our previously described (Chen et al., 2017). Overnight cultures of *E. coli* strains were diluted to 10^9^ cfu/mL into CAMHB and grown in the same medium for 20 min with 10 mg/L of tigecycline alone and in combination with 2 mg/L of colistin. Bacterial cells were collected by centrifugation at 3000 × *g* for 10 min, washed with sterile normal saline and dried to obtain the dry weight. Bacteria cells were lysed by sonication for 15 min and then centrifuged at 3000 × *g* for 10 min to remove the cell debris. Tigecycline concentrations in the resulting cell extracts were determined by a LC-MS/MS method (Sun et al., 2019; details are given in the Supplementary Material). All experiments were performed at least five independent biological replicates. Results were expressed as amount of tigecycline incorporated per dry weight of bacteria.

### *In vitro* Time-Kill Experiments

*In vitro* time-kill experiments were conducted to characterize the activity of the colistin and tigecycline combination using previously described methods (Rao et al., 2016). In brief, overnight *E. coli* cultures (∼10^6^ or 10^8^ cfu/mL) were exposed to colistin (2 and 8 mg/L) alone and in combination with tigecycline (0.25 mg/L) over a period of 48 h. The choice of colistin concentrations was based on the clinically achievable serum steady-state concentration (C_ss_) and peak concentration (C_*max*_) in humans, while the tigecycline concentration was chosen to simulate the average C_ss_ at the clinical dose of 50 mg every 12 h (Van Wart et al., 2006; Tran et al., 2016; Nation et al., 2017). Serial samples were obtained at 0, 1, 3, 6, 9, 12, 24, 27, 30, 33, and 48 h after incubation at 37°C. Bacterial counts were determined based on the quantitative cultures on MHA plates. Historical time-kill data of colistin alone for portion of study strains were obtained from our previous report (Zhou et al., 2017).

### *In vitro* Pharmacodynamic (PD) Analysis

The concentration-effect curves were used to quantitatively evaluate the potency of colistin and tigecycline combination against *E. coli* strains harboring *bla*_NDM–__5_ and *mcr-1*, at initial inoculums of 10^6^ and 10^8^ cfu/mL, respectively. The testing procedure consisted of four groups, and each group included tubes with twofold increasing concentrations of colistin from 0.5 to 16 mg/L, in the absence and presence of tigecycline at 0.13, 0.25, and 0.5 mg/L. After 48 h of incubation, the microbiological response was measured by the log_10_ change in bacterial density vs. pre-exposure at 0 h. The relationships between colistin concentrations and antibacterial response to single and combination therapies were fit to the Hill sigmoid E_*max*_ model: E = E_0_ + E_*max*_ × C^N^/(EC_50_^N^ + C^N^), where E_0_ is the log_10_ change in bacterial count without colistin, E_*max*_ is the maximal effect, EC_50_ is the colistin concentration required to achieve 50% of E_*max*_ and N is the slope of concentration-effect curve. The PD analysis was carried out by the non-linear least-squares regression in WinNonlin software Version 6.1 (Pharsight, Sunnyvale, CA, United States) (Zhou et al., 2017). The coefficient of determination (*R*^2^) was used to estimate the variance of PD regression analysis. Mann-Whitney test was used to compare the parameters of E_*max*_ and EC_50_ between *mcr-1*-positive and -negative strains. Differences of PD parameter at 10^6^ vs. 10^8^ cfu/mL inoculum were determined using Wilcoxon signed-rank test in GraphPad Prism 8 software (San Diego, CA, United States) and a *P* value of <0.05 was considered significant.

### Murine Thigh Infection Model and Treatment Regimens

All animal experimental protocols were approved by South China Agricultural University (SCAU) Institutional Animal Ethics Committee (Guangzhou, China) and performed in accordance with the SCAU Institutional Laboratory Animal Care and Use guidelines. Six-week-old, 25–27 g, specific-pathogen-free, female ICR mice (Hunan SJA Laboratory Animal, Changsha, China) were rendered neutropenic by administration of cyclophosphamide intraperitoneally as previously described (Zhou et al., 2018). Thigh infections with each *E. coli* were produced by injecting 0.1 mL of bacterial suspension in normal saline (10^6.5^ and 10^8.5^ cfu/mL). At 2 h after infection, mice were randomized to receive (i) no therapy (control), (ii) colistin at 7.5 mg/kg intraperitoneally (i.p.) twice a day (bid), (iii) tigecycline at 5 mg/kg subcutaneously (s.c.) bid, or (iv) combination of colistin and tigecycline. The current usual doses of colistin (3 MIU, equivalent to 240 mg, every 8 h) and tigecycline (100 mg initially, then 50 mg bid) were acceptable for the treatment of severe infections in humans (Meagher et al., 2005; Docobo-Perez et al., 2012). In this study, the drug doses in mice were selected to mimic the pharmacokinetic profiles of human clinical doses of 300 and 200 mg, respectively (Meagher et al., 2005; Karnik et al., 2013; Zhou et al., 2017; Zhao et al., 2018). Control and antibiotic-treated mice were sacrificed at 24 h after start of therapy. Thigh muscles were aseptically removed, homogenized and bacteria were cultured quantitatively using the plate counting method, and results were expressed as the log_10_ cfu/thigh. Three mice (i.e., six thighs) were included in each group. The Mann-Whitney *U*-test was used to compare bacterial densities in target tissue between mono- and combination therapies.

## Results

### *In vitro* Susceptibility and Interaction Assessment

**FIGURE 2:**
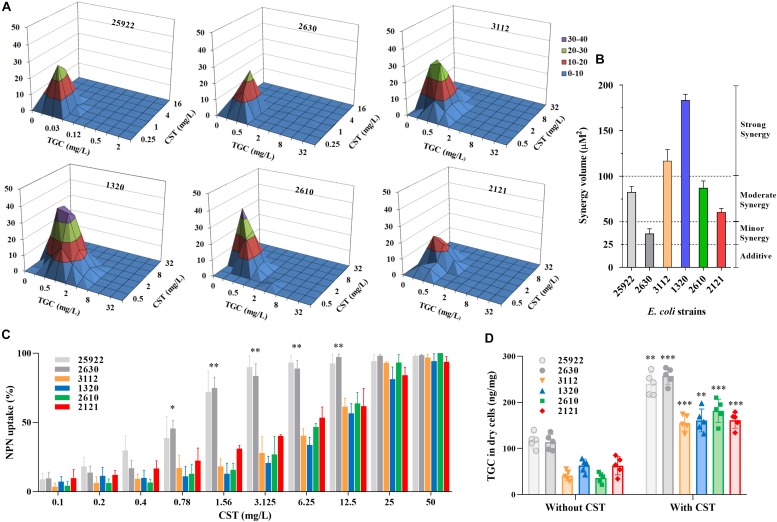
*In vitro* interactions between colistin and tigecycline. **(A,B)** Synergism as demonstrated using MacSynergy II plots of the three-dimensional dose-response curves. The flat plane represents the predicted indifference between antagonism and synergy. Peaks and troughs represent synergy and antagonism, respectively. Synergy expressed as the calculated interaction volumes (μM^2^) at a confidence interval of 95%: <25, additive; 25 to 50, minor but significant; 50 to 100, moderate; and >100, strong synergy. **(C)** Colistin-induced NPN uptake (%) of *mcr-1*-positive and -negative *E. coli* strains. The data represents background subtracted fluorescence divided by the fluorescence observed at 100 mg/L of colistin. **(D)** Accumulations of tigecycline in *E. coli* strains (dry weight) after exposure to 10 mg/L tigecycline for 20 min in the presence and absence of colistin. Data shown are the means of five independent biological replicates. ^∗^*P* < 0.05; ^∗∗^*P* < 0.01; and ^∗∗∗^*P* < 0.001.

The carbapenem-resistant *E. coli* strains were highly resistant to almost all tested antibiotics (Table 1). As expected, *E. coli* strain 2630 lacking *mcr-1* was susceptible to colistin, with an MIC of 0.5 mg/L in the absence of tigecycline (Table 1). However, the strains that harbored *bla*_NDM–__5_ and *mcr-1* were resistant both to meropenem (MIC ≥ 16 mg/L) and colistin (MIC ≥ 4 mg/L). Interestingly, colistin MICs for *mcr-1*-positive CRE strains decreased to 1/4 to 1/16 of the original levels as tigecycline concentration was raised from 0 to 0.5 mg/L (Table 1). This was confirmed using the checkerboard assay that showed synergistic effects of the colistin and tigecycline combination. The FICI values varied from 0.38 to 0.5 for all except the colistin susceptible strain 2630 (Table 1). In particular, *E. coli* 1320 that carried both *bla*_NDM–__5_ and *mcr-1* displayed a highly significant synergistic response to this combination across the range of drug concentrations tested, with a clear peak at 0.5 mg/L tigecycline and 1 or 2 mg/L colistin (Figure 2A). Different degrees of synergy were observed for all study *E. coli* strains with synergy volumes that ranged from 36.9 to 183 μM^2^ (Figure 2B).

**TABLE 1:** Genotype summary, *in vitro* antimicrobial susceptibility profiles, and MICs of colistin in the absence and presence of tigecycline at 0.13, 0.25, and 0.5 mg/L against study *E. coli* strains*^*a*^*.

***E. coli* strain**	**Relevant genotype**	**MIC (mg/L)**								**CST MIC (mg/L)**				**FIC index**
		**AMP**	**CTX**	**MEM**	**GEN**	**CIP**	**RIF**	**TET**	**TGC**	**CST alone**	**TGC 0.13**	**TGC 0.25**	**TGC 0.5**	
25922	ST73; ATCC strain	4	0.06	0.03	0.5	0.008	4	1	0.13	1	NA	NA	NA	0.5
2630	ST3902; *bla*_NDM–5_	256	256	64	16	256	32	128	1	0.5	0.5	0.25	0.13	0.75
3112	ST1011; *mcr-1*	256	128	0.13	256	128	256	64	1	8	2	2	1	0.37
1320	ST648; *bla*_NDM–5_/*mcr-1*	128	64	16	32	128	8	128	2	4	2	0.5	0.5	0.37
2610	ST101; *bla*_NDM–5_/*mcr-1*	256	256	16	64	256	256	64	1	4	2	1	1	0.5
2121	ST156; *bla*_NDM–5_/*mcr-1*	256	256	16	128	128	4	128	1	8	4	2	0.5	0.5

*^*a*^AMP, ampicillin; CTX, cefotaxime; MEM, meropenem; GEN, gentamicin; CIP, ciprofloxacin; RIF, rifampicin; TET, tetracycline; TGC, tigecycline; CST, colistin; NA, not applicable.*

### Colistin-Induced Outer-Membrane Perturbation and Tigecycline Accumulation

**FIGURE 3:**
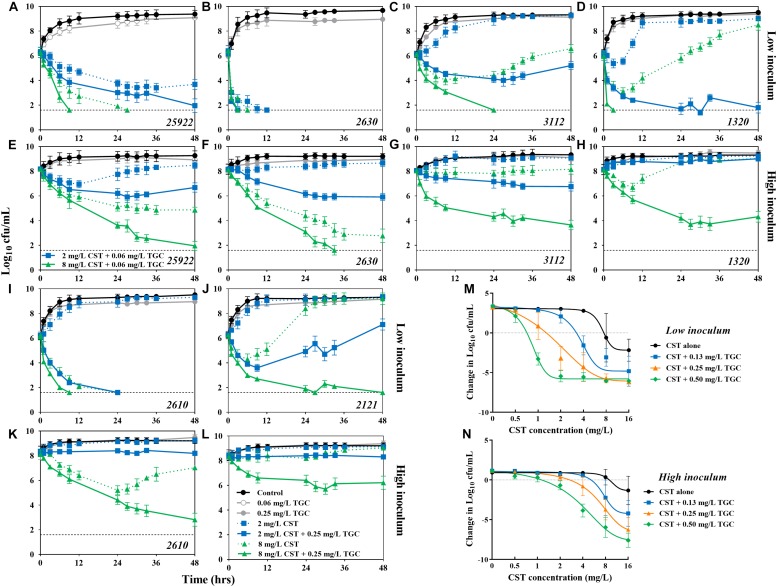
Combinatorial bactericidal activity of colistin and tigecycline against *mcr-1*-positive and -negative *E. coli* strains harboring *bla*_NDM–5_. **(A–L)** *In vitro* time-kill experiments of colistin (2 and 8 mg/L) alone and in combination with tigecycline (0.25 mg/L) against all study *E. coli* strains at low and high inoculums over 48 h. The horizontal dotted line represents the limit of detection for bacterial count (40 cfu/mL). Historical time-kill data of colistin alone for potion of strains was obtained from our previous study (Zhou et al., 2017). **(M,N)** The concentration-effect profiles of colistin against *E. coli* strains harboring both *bla*_NDM–5_ and *mcr-1* (i.e., 1320, 2610, and 2121) at low **(M)** and high **(N)** inoculums following treatment with colistin (0–16 mg/L) at fixed concentrations of tigecycline (0–0.5 mg/L). Each symbol represents the log_10_ change in bacterial burdens over a 48 h study period. Data points below the line represent killing and points above the line represent growth.

Carriage of *mcr-1* in carbapenem-resistant *E. coli* strains increased their resistance to colistin-induced outer-membrane disruption as expected. NPN uptake in *mcr-1*-harboring *E. coli* was significantly less than *E. coli* 2630 after exposure to colistin at 0.78 to 12.5 mg/L (Figure 2C; *P* < 0.05), with corresponding colistin MIC increases from 8- to 16-fold (Table 1). The colistin concentrations required to achieve the comparable levels of NPN uptake increased eightfold in mcr*-1*-positive compared to -negative *E. coli* strains. For example, 45% of NPN uptake was observed at 0.78 mg/L colistin for colistin-susceptible *E. coli* 2630, while similar NPN uptake (38% to 53%) occurred at 6.25 mg/L colistin for *mcr-1*-harboring strains (Figure 2C). It seems that the additional levels of outer-membrane perturbation in a colistin-susceptible strain can be achieved by increasing the concentration of colistin eightfold in *mcr-1*-harboring *E. coli* strains. Importantly, when combined with the clinically relevant concentration of colistin at 2 mg/L, intracellular accumulations of tigecycline markedly increased in all study *E. coli* strains (*P* < 0.01; Figure 2D). Although the concentration of 2 mg/L colistin alone was insufficient to inhibit growth of *E. coli* harboring both *bla*_NDM–__5_ and *mcr-1* (Figures 3H-J), it provided sufficient outer-membrane perturbation to facilitate tigecycline uptake and subsequent tigecycline-induced growth inhibition (Figure 2D).

### *In vitro* Time-Kill Experiments

At a low inoculum (10^6^ cfu/mL), colistin alone at 2 mg/L achieved complete the bactericidal activity (>6.3-log_10_ reduction) over 24 h against colistin-susceptible strain 2630. The activity was not further improved at higher colistin levels or in combination with tigecycline (Figure 3B). Against the colistin-resistant *E. coli* 1320, the clinically achievable concentrations of colistin resulted in early bactericidal activity only, with a 1.3- to 3.2-log_10_ reduction in bacterial density, followed by rapid regrowth beyond 6 h. However, complete bacterial eradication was attained with the combination of 8 mg/L colistin and 0.25 mg/L tigecycline (Figure 3D). Similarly, in the presence of 0.25 mg/L tigecycline, substantial killing of *E. coli* 2610 was achieved with >2 mg/L colistin (Figure 3I). Interestingly, despite the lack of activity that was observed for all colistin monotherapies against *E. coli* 2121, tigecycline displayed the ability to increase killing activity over 48 h of exposure to colistin (Figure 3J).

Monotherapy with a high colistin concentration (8 mg/L) or the combination of 0.25 mg/L tigecycline and 2 mg/L colistin exhibited sustained bactericidal activity at the high inoculum (10^8^ cfu/mL) of *E. coli* 2630 (Figure 3F). However, even the high colistin levels of 8 mg/L were inactive for the colistin-resistant strains, whereas in combination with 0.25 mg/L tigecycline resulted in a 2.1- to 3.9-log_10_ reduction in bacterial density (Figures 3H,K–L). Tigecycline monotherapy at 0.06 or 0.25 mg/L performed no different from the growth control against all study *E. coli* at both low and high inoculums (Figure 3).

### Concentration-Effect Relationships

The concentration-effect relationship was fitted to a Hill-type equation (*R*^2^ > 0.95), and the PD parameter of EC_50_ representing colistin potency was significantly greater in *mcr-1*-harboring strains compared with *E. coli* 2630 (*P* < 0.01; Table 2). In addition, the EC_50_ values at 10^8^ cfu/mL inoculum were 1.5- to 18.4-times higher than those at 10^6^ cfu/mL inoculum (mean = 5.3, *P* < 0.001). In the three strains that harbored *bla*_NDM–__5_ and *mcr-1*, a clear tendency toward higher E_*max*_ values were seen with a 10^8^ cfu/mL inoculum, whereas no significant difference was noted at 10^6^ cfu/mL (Table 2).

**TABLE 2:** Hill PD parameters describing the concentration-response profiles of colistin (0–16 mg/L) in the presence of fixed tigecycline concentrations (0–0.5 mg/L) at low and high inoculums*^*a*^*.

**TGC (mg/L) in combination**	**10^6^ cfu/mL**			**10^8^ cfu/mL**		
	**E_*max*_**	**EC_50_**	***N***	**E_*max*_**	**EC_50_**	***N***
**PD parameters for *E. coli* ATCC 25922**						
0	−9.61	1.58	2.24	−6.46	6.19	1.94
0.03	−9.85	1.01	1.53	−8.95	4.95	1.12
0.06	−8.74	0.83	1.30	−9.09	3.47	1.40
0.13	−7.22	0.55	1.73	−9.13	2.01	1.43
**PD parameters for *E. coli* carrying *bla*_NDM–__5_ (i.e., isolate 2630)**						
0	−9.43	0.82	8.13	−9.11	6.75	4.68
0.13	−9.68	0.49	10.7	−9.14	5.45	3.71
0.25	−9.56	0.27	10.1	−9.17	4.98	1.18
0.50	−9.57	0.26	12.9	−9.41	2.36	1.22
**PD parameters for *E. coli* carrying *mcr-1* only (i.e., isolate 3112)**						
0	−5.60	5.98	2.07	−2.28	8.68	1.04
0.13	−9.48	3.96	2.55	−4.33	6.01	1.71
0.25	−9.42	2.34	2.37	−7.05	3.55	1.49
0.50	−9.30	1.05	1.84	−7.52	3.48	1.59
**Mean PD parameters for *E. coli* carrying *bla*_NDM–__5_ and *mcr-1* (i.e., 1320, 2610, and 2121)**						
0	−6.53	7.37	5.66	−2.31	10.6	4.34
0.13	−8.23	5.50	3.97	−5.25	9.91	3.69
0.25	−9.42	2.09	2.56	−7.49	7.02	2.83
0.50	−9.36	0.80	4.17	−8.60	3.98	2.13

*^*a*^E_*max*_, maximum effect compared to the no drug control for a log_10_ change of bacterial density after the 48 h study period; EC_50_, colistin concentration required to achieve 50% E_*max*_; N, slope of the concentration-effect curve.*

Overall, we found similar dose-dependent shifts with increasing tigecycline levels to a lower colistin concentration required to suppress the growth of *E. coli* at both inoculums (Figures 3M,N). For example, at 10^6^ cfu/mL, inhibition of *E. coli* 2630 occurred at the colistin concentration of 0.75 mg/L and decreased threefold to 0.25 mg/L in the presence of tigecycline (Supplementary Figure S1C). Carriage of *mcr-1* increased the colistin concentration required for growth inhibition to 8 mg/L, which was 11-fold greater than for *E. coli* 2630 (Figure 3M). However, in combination with tigecycline from 0.13 to 0.5 mg/L, the colistin levels for growth inhibition were only 0.75 mg/L or twofold and fourfold greater than the concentration needed to synergize with tigecycline against *E. coli* 2630 (Figure 3M and Supplementary Figure S1C). It seems that the *mcr-1* gene only provided protection against colistin monotherapy, but not an ability to resist the colistin and tigecycline combination therapy.

### *In vivo* Efficacy of Mono- and Combination Therapies

**FIGURE 4:**
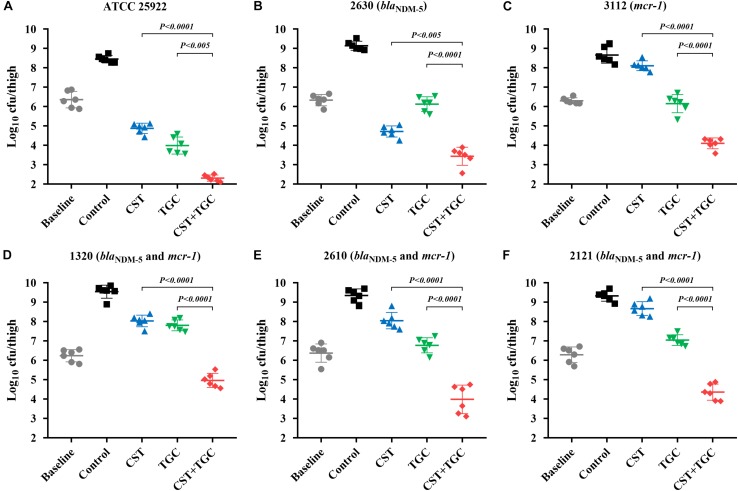
Efficacy of colistin and tigecycline mono- and combination therapies at 24 h against *E. coli* ATCC 25922 **(A)**, 2630 **(B)**, 3112 **(C)** and strains harboring *bla*_NDM–5_ and *mcr-1* **(D–F)** in the murine thigh infection model with a low initial burden of 10^6^ cfu/thigh. Colistin (7.5 mg/kg i.p. bid) and tigecycline (5 mg/kg s.c. bid) and the combination were administrated at 2 h post-infection. Horizontal lines represent the mean and standard deviation of bacterial densities for each group (*n* = 6). Colistin and tigecycline combination therapy resulted in a >2.0 log_10_cfu/thigh reduction relative to each monotherapy (*P* < 0.0001, Mann-Whitney *U*-test).

**FIGURE 5:**
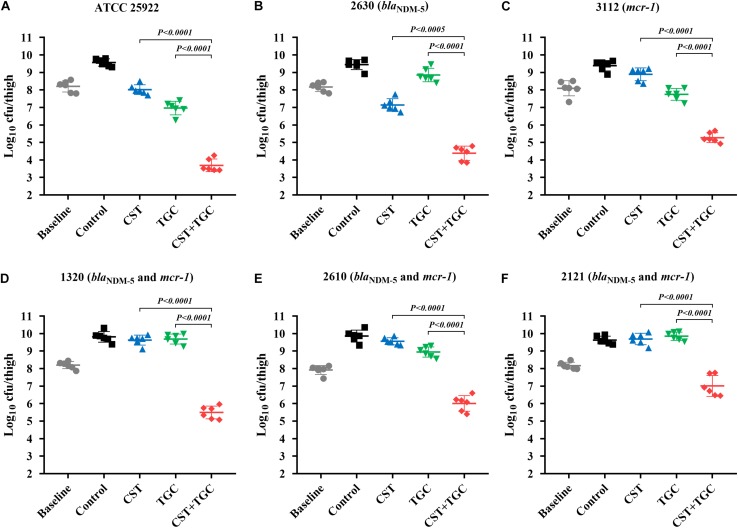
Efficacy of colistin and tigecycline mono- and combination therapies at 24 h against *E. coli* ATCC 25922 **(A)**, 2630 **(B)**, 3112 **(C)** and strains harboring *bla*_NDM–5_ and *mcr-1* **(D–F)** in the murine thigh infection model with a high initial burden of 10^8^ cfu/thigh. Colistin (7.5 mg/kg i.p. bid) and tigecycline (5 mg/kg s.c. bid) and the combination were administrated at 2 h post-infection. Horizontal lines represent the mean and standard deviation of bacterial densities for each group (*n* = 6). Colistin and tigecycline combination therapy resulted in a >2.5 log_10_cfu/thigh reduction relative to each monotherapy (*P* < 0.0005, Mann-Whitney *U*-test).

During thigh infection with a low initial burden, colistin monotherapy led to decreased bacterial density by 1.62-log_10_cfu/thigh for colistin-susceptible *E. coli* 2630, compared to the untreated control at 0 h (Figure 4B). However, for colistin-resistant strains, neither colistin nor tigecycline monotherapy showed a significant reduction in bacterial density after 24 h of therapy. Interestingly, colistin and tigecycline combination proved efficacious, resulting in >2.0 log_10_cfu/thigh reduction compared to each monotherapy (*P* < 0.0001, Mann-Whitney *U*-test; Figures 4C–F). The high initial burden in the murine thigh infection model was used to stimulate the severe infections that result in high mortality, and the effectiveness of combination therapy is a general proof of principle. Monotherapy with colistin or tigecycline did not achieve notable antibacterial effects against *E. coli* harboring *bla*_NDM–__5_ and *mcr-1* at the high initial inoculum (Figure 5). Importantly, the combination of colistin and tigecycline significantly increased killing activity at 24 h by 1.1- to 2.7- log_10_cfu/thigh reduction in bacterial density compared to control at 0 h or >2.5-log_10_cfu/thigh compared to each monotherapy (*P* < 0.0005, Mann-Whitney *U*-test; Figures 5D–F).

## Discussion

Treatment options for carbapenem-resistant *E. coli* infections are very limited especially if the *mcr-1* gene is also present in the infecting strains. Tigecycline and colistin are currently the last-resort antibiotics for the treatment of severe infections (Sun et al., 2019). However, tigecycline demonstrates mainly bacteriostatic activity with low serum levels (Van Wart et al., 2006). Concerns have been raised regarding the efficacy of tigecycline monotherapy in the light of decreased clinical success rates (Yahav et al., 2011). Indeed, in the current study, tigecycline monotherapy did not achieve positive outcomes in a murine thigh infection model when the study *E. coli* strains harbored both *bla*_NDM–__5_ and *mcr-1*, despite the fact that most of strains (5/6) remained susceptible to tigecycline except the strain 1320. Fortunately, the presence of *mcr-1* only slightly increased the MIC of colistin (Zhou et al., 2017). Consequently, there was a compelling reason to use colistin and tigecycline in combination.

Colistin and tigecycline combination therapy against CRE infection had varying outcomes from synergy to indifference (Bercot et al., 2011; Karaoglan et al., 2013; Rao et al., 2016; Cai et al., 2017; Ku et al., 2017). In this study, combination of clinically achievable concentration of colistin and tigecycline produced a synergistic activity *in vitro* against *E. coli* harboring *bla*_NDM–__5_ and *mcr-1*, resulting in a >4.0-log_10_cfu/mL reduction by 48 h. An additional *in vivo* synergistic effect was indeed observed in the murine thigh model, at both low and high inoculums. Supporting our findings, colistin displayed a similar synergistic interaction with tigecycline for carbapenem-resistant *A. baumannii* and *K. pneumoniae* (Pournaras et al., 2011; Karaoglan et al., 2013; Ku et al., 2017). Data from previous case reports also showed beneficial activity of tigecycline and colistin combination therapy against *K. pneumoniae* bacteremia (Cobo et al., 2008). Interestingly, the higher dose of tigecycline has been shown to be associated with better synergistic outcomes against multidrug-resistant CRE, compared with the conventional dosing regimen (De Pascale et al., 2014; Cai et al., 2017). In contrast, a potential trend toward antagonism was observed at lower tigecycline concentrations (Albur et al., 2012). Of note, previous studies that used this combination employed different methods, and the isolates were not well-described genotypically, making the results difficult to generalize. Here, we demonstrated increased activity of colistin in combination tigecycline against *E. coli* strains that harbored *bla*_NDM–__5_ and *mcr-1*, including the pandemic clonal complex ST648 (Hornsey et al., 2011). The clinical impact of infections due to colistin-resistant NDM-5-producing *E. coli* is currently unknown, but our findings provide an alternative approach to combat such resistant strains. In support of this view, a recent report indicated that colistin and tigecycline combination was able to prevent the emergence of high-level resistance to these antibiotics (Cai et al., 2017).

The potentiation effect of this combination is most likely related to their different mechanisms of action at separate bacterial targets. Tigecycline acts in the cytoplasm by binding to the ribosomal complex that requires drug to enter the bacterial cells first (Bauer et al., 2004). In general, uptake of tigecycline across the bacterial cell wall and cytoplasmic membrane includes two ways: passive diffusion and an energy-dependent active transport system (Schnappinger and Hillen, 1996; Chopra and Roberts, 2001). In Gram-negative bacteria, the cell wall is surrounded by the outer-membrane and tigecycline moves through membranes via porin channels in the absence of colistin (Roberts, 2003). Colistin resulted in bacterial outer-membrane disruption and instable regions in cytoplasmic membrane that may facilitate tigecycline passive accumulation (Macnair et al., 2018). Supporting this speculation, our NPN uptake and intracellular tigecycline accumulation assays demonstrated that exposure to colistin did promote tigecycline uptake and subsequent tigecycline-induced growth inhibition independent of *mcr-1* expression. This scenario has been reported for colistin in combination with minocycline, the prodrug of tigecycline (Liang et al., 2011). However, the precise details of how colistin affects the energy-dependent transport of tigecycline still remain unclear.

Owing to the paucity of novel antibiotics, colistin-based combination therapy was therefore regarded as an alternative approach to combat colistin-resistant CRE infections. A synergistic effect of colistin with amikacin, rifampicin, and osthole has been reported (Lagerback et al., 2016; Liu X. et al., 2016; Zhou et al., 2017, 2019). However, systemic administration of colistin is associated with nephrotoxicity despite the fact that toxicity is dose-dependent and reversible on discontinuation of treatment (Biswas et al., 2012). Therefore, the clinical utility of colistin should be prudent when used in combination with other nephrotoxic antibiotics such as gentamicin and amikacin. Previous nephrotoxicity studies in mice indicated that only mild kidney damage was observed until an accumulated dose of 72 mg/kg colistin, and suggested an acceptable colistin single dose ranges within 40 mg/kg in mice (Cheah et al., 2015; Roberts et al., 2015). Therefore, the much lower colistin dose (7.5 mg/kg) that used in this study should be safe for mice by comparison. In fact, many previous studies have employed 7.5 mg/kg colistin to carry out *in vivo* efficacy studies in mice (Liu et al., 2016; Zhou et al., 2017; Macnair et al., 2018). In the present study, tigecycline demonstrated bactericidal activity against *E. coli* harboring *bla*_NDM–__5_ and *mcr-1* when combined with the clinically relevant concentration of colistin at 2 mg/L, which is considered as the appropriate partnered concentration to avoid renal impairment (Tran et al., 2016). Importantly, the combination of tigecycline with colistin we studied here may allow lower colistin dose sparing regimens that reduce nephrotoxicity for treating colistin-resistant CRE infections. Previous comparative observational studies also showed a lower-than-expected toxicity for tigecycline and colistin combination therapy (Zhang et al., 2013). Even patients with kidney disease could benefit from colistin-based combination therapy, when provided with a lower daily dose of colistin achieving comparable efficacy (Falagas et al., 2006; Biswas et al., 2012). In addition, a retrospective cohort study indicated that colistin is a valuable antibiotic with acceptable nephrotoxicity (<7%) and considerable efficacy that depends on daily dose (Falagas et al., 2010).

Our investigation has several limitations. For example, the combination was evaluated in a small number of strains despite the different clonal types. In addition, the murine thigh model is a local infection model, and additional study is needed to evaluate the usefulness of this combination in the clinical setting. Moreover, based on our current results, we do not know whether the colistin-induced increased accumulation of tigecycline in bacterial cells is “drug specific” or more broad range for other antibiotics. Although this is beyond the scope of this study, future studies should examine this potential mechanism.

In summary, this study demonstrated increased activity of colistin and tigecycline combination against *E. coli* harboring *bla*_NDM–__5_ and *mcr-1*. Importantly, a potentiation effect occurred at the clinically relevant concentrations of colistin and tigecycline, and was efficacious in the murine thigh infection model. In addition, we demonstrated for the first time that colistin permeabilization of the bacterial outer-membrane facilitates the uptake of tigecycline, contributing to increased activity of the combination.

## Data Availability Statement

The raw data supporting the conclusions of this article will be made available by the authors, without undue reservation, to any qualified researcher.

## Ethics Statement

This study was carried out in accordance with the recommendations of ethical guidelines of South China Agricultural University. All animal experimental protocols and isolation procedures for *E. coli* strains were reviewed and approved by the South China Agricultural University Institutional Animal Ethics Committee (2019B161 and 2018B095). Individual written informed consent for the use of isolates was obtained.

## Author Contributions

Y-HL and Y-FZ designed the study and wrote the manuscript. Y-FZ, PL, and C-JZ carried out the experiments. JS and X-PL analyzed the data. All authors read and approved the final manuscript.

## Conflict of Interest

The authors declare that the research was conducted in the absence of any commercial or financial relationships that could be construed as a potential conflict of interest.
