# Supplementary material for: Colistin Combined With Tigecycline: A Promising Alternative Strategy to Combat Escherichia coli Harboring blaNDM–5 and mcr-1
Source: Front Microbiol. 2020 Jan 8;10:2957. doi: 10.3389/fmicb.2019.02957 (PMC6960404; doi:10.3389/fmicb.2019.02957)
Supplement: Supplementary file 1 [file Data_Sheet_1.pdf]

Supplementary Materials

Sample processing and LC-MS/MS method

A 0.5 mL aliquot of bacterial cell lysates was transferred into a centrifuge tube, and then mixed with 0.5 mL of acetonitrile. After vortexing (1 min) and centrifuging (12000×g, 10 min), the supernatant was filtered through a 0.22 μm nylon syringe filter and collected into a sample vial for concentration determination. The concentrations of tigecycline were determined using a HPLC-ESI-MS/MS system (Agilent 1200 HPLC system; API 4000 triple quadrupole mass spectrometer; Applied Biosystems, Carlsbad, CA, USA) equipped with a C_{18} column (Waters Symmetry, 2.1×100 mm, 3.5 μm). The injection volume was 5 μL, and column temperature was maintained at 30°C. Mobile phase consisted of (A) acetonitrile and (B) 0.1% formic acid in water containing 10 mM ammonium formate using a gradient elution with a flow rate of 200 μL/min: 0-0.5 min (5% A), 0.5-1.0 min (5-95% A), 1.0-3.0 min (95% A), 3.0-3.5 min (95-5% A), 3.5-10 min (5% A). The total run time was 10 min. Mass spectrometer was carried out in positive ESI mode as follows: ionspray voltage, 5000 V; curtain gas, 30 psi; collision gas, 5 psi; source temperature, 550 °C. Quantification was performed using selected reaction monitoring of ion transitions of m/z 586.3→513.4 for tigecycline, with the declustering potential and collision energy of 100 and 35 V, respectively.

Calibration standards and quality control samples were prepared in pooled lysates of bacterial cells. Calibration standards in the range of 10-500 ng/mL were prepared by spiking appropriate amount of stock solutions into the blank bacterial cell lysate matrix. The correlation coefficients (R) were >0.995 in the linear range of 10-500 ng/mL. All samples that had concentrations >500 ng/mL were diluted proportionally with blank lysate matrix. The limit of quantification (LOQ) and detection (LOD) were 10 and 5 ng/mL, respectively. The accuracy ranged from 87.4 to 106.8%, and intra- and inter-batch precision were <8.09% at all tested concentrations.

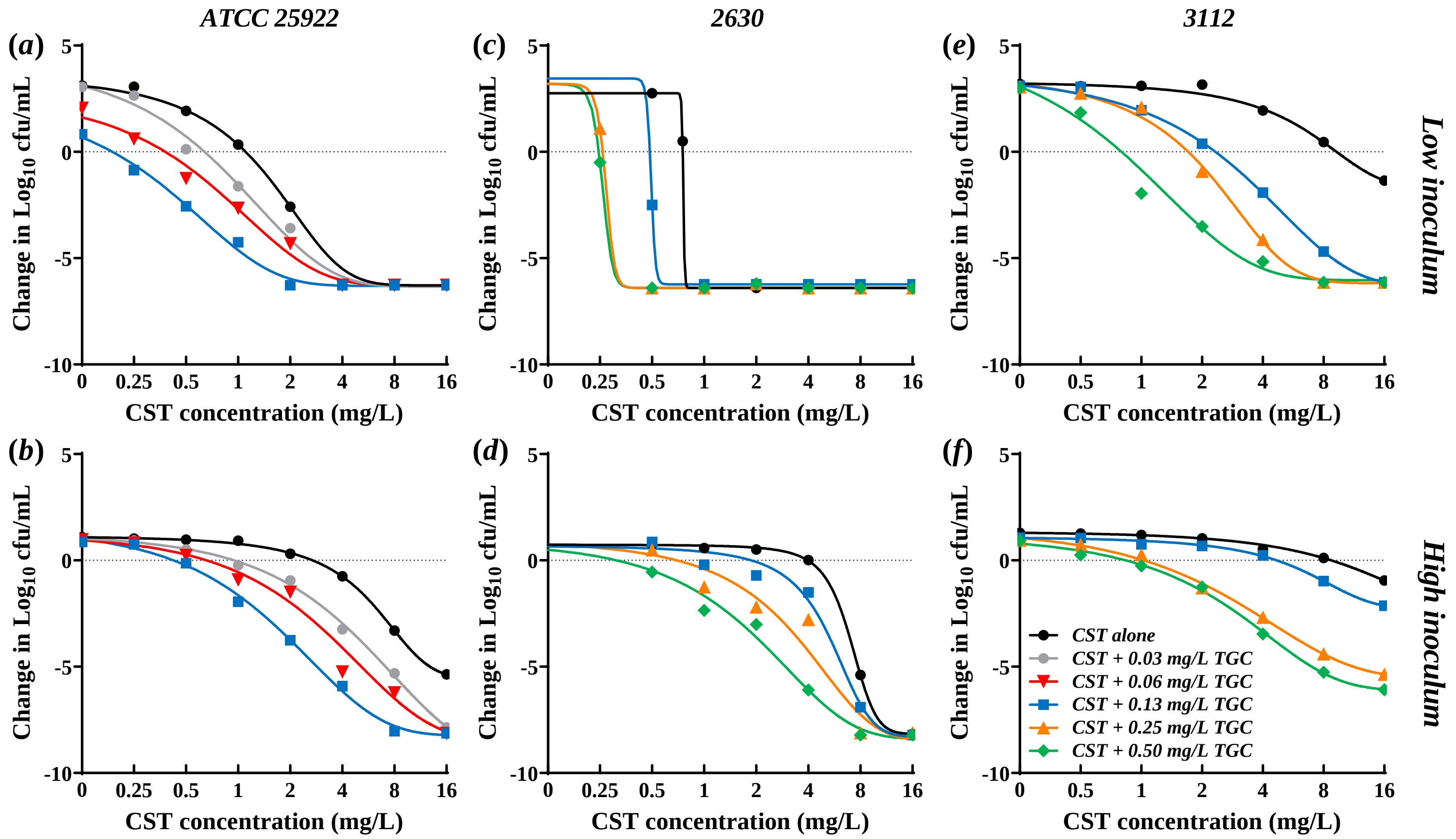

Figure S1. The concentration-effect profiles of colistin for *E. coli* ATCC 25922 (***a, b***), *bla*$_{\text{NDM-5}}$-carrying *E. coli* strain 2630 (***c, d***) and *mcr-1*-carrying *E. coli* 3112 (***e, f***) at low (upper panels) and high (lower panels) inoculums, following exposure to colistin (0-16 mg/L) at fixed tigecycline concentrations (0-0.5 mg/L). Each symbol represents the $\log_{10}$ change in bacterial burdens over the 48 h study period. Data points below the line represent killing and points above the line represent growth.
